# Supplementary material for: Evolution of sexual size dimorphism in the wing musculature of Drosophila
Source: PeerJ. 2020 Jan 17;8:e8360. doi: 10.7717/peerj.8360 (PMC6970592; doi:10.7717/peerj.8360)
Supplement: Supplemental Information 1 [file peerj-08-8360-s001.pdf]

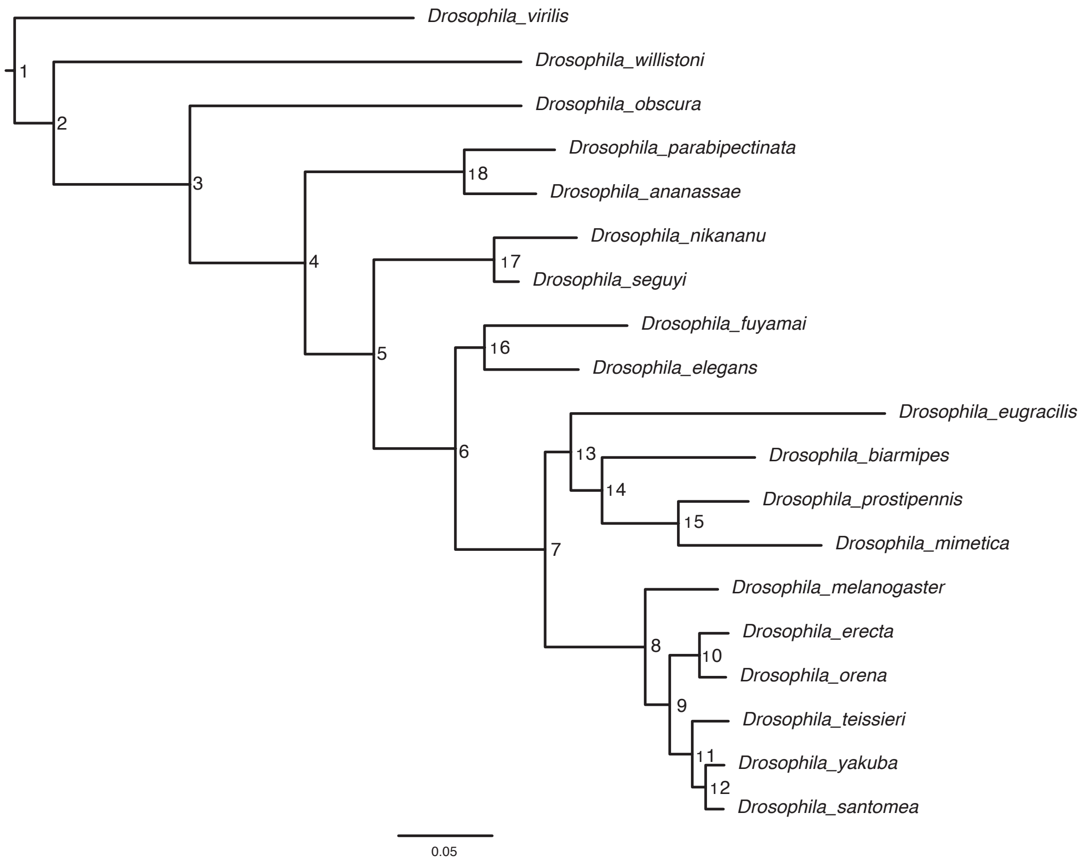

Supplemental Figure 1. Maximum likelihood tree showing phylogenetic relationships among 19 Drosophila species. Each ancestral node is numbered. Probabilities of a monomorphic or dimorphic ancestor at each node is shown in Supplemental Table 1.

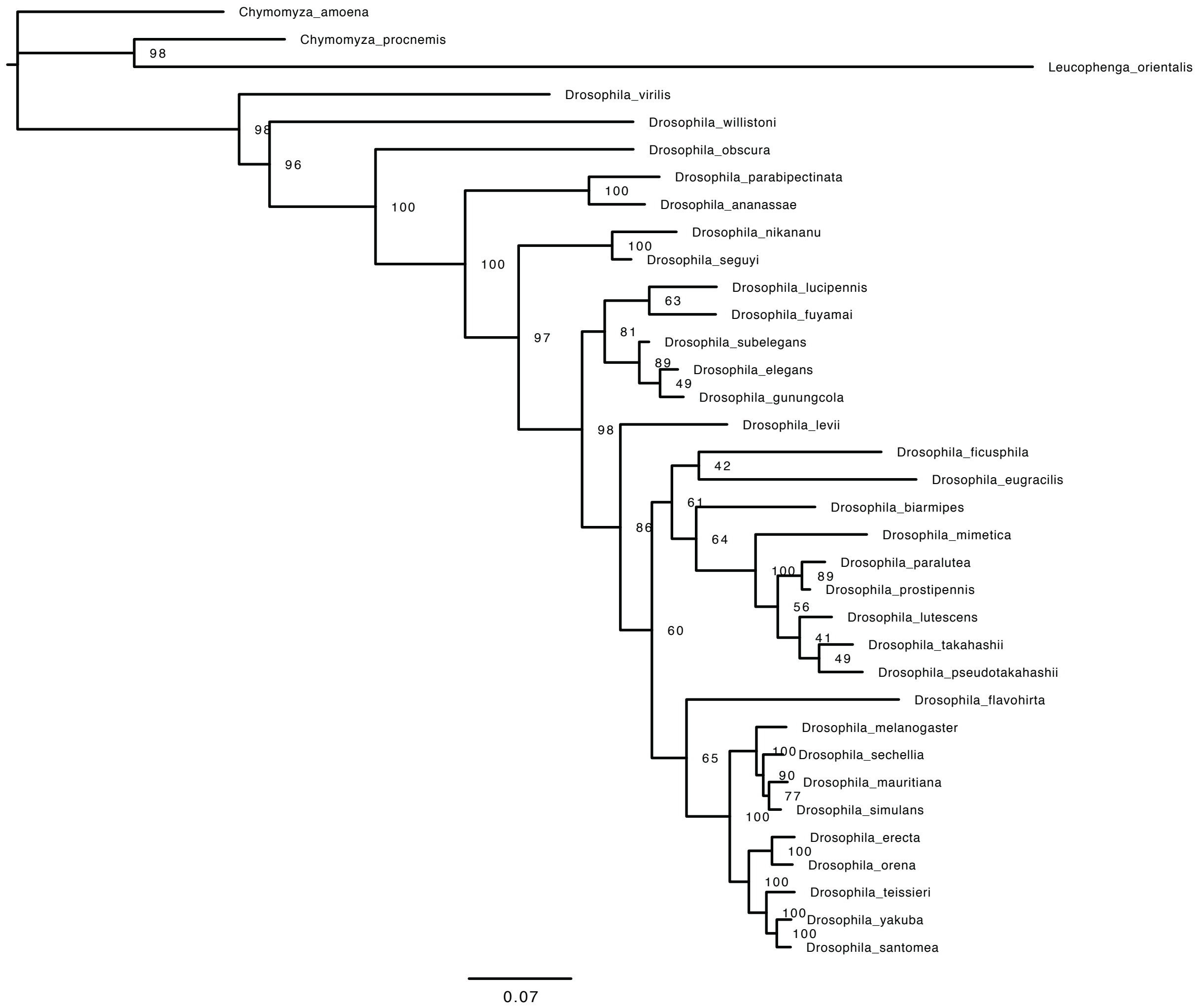

Supplemental Figure 2. Complete phylogenetic estimate from Maximum Likelihood analysis of all six nuclear genes concatenated (ADH, amyrel, Ddc, Gphd, Sod, and Xdh). Node labels indicate ultrafast bootstrap support values, with a value of 95 or greater indicating a well supported node.

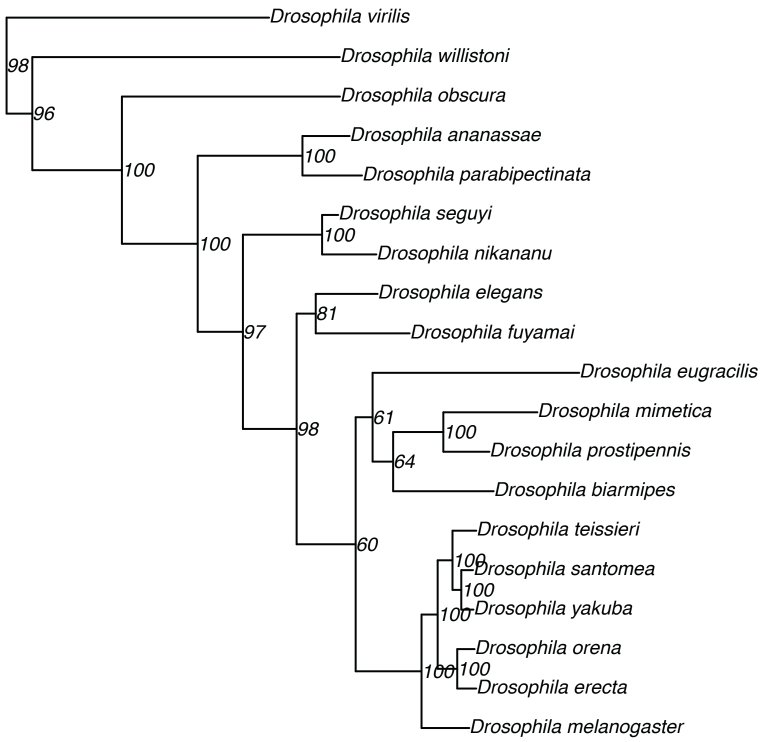

Supplemental Figure 3. Paired down phylogeny used for Ancestral State Reconstructions seen in Figures 3-4. Full phylogeny (Supplemental Figure 2) was paired down to include only species for which muscle volume data was available for the ancestral state reconstruction. Node labels indicate ultrafast bootstrap support values, with a value of 95 or greater indicating a well supported node. a

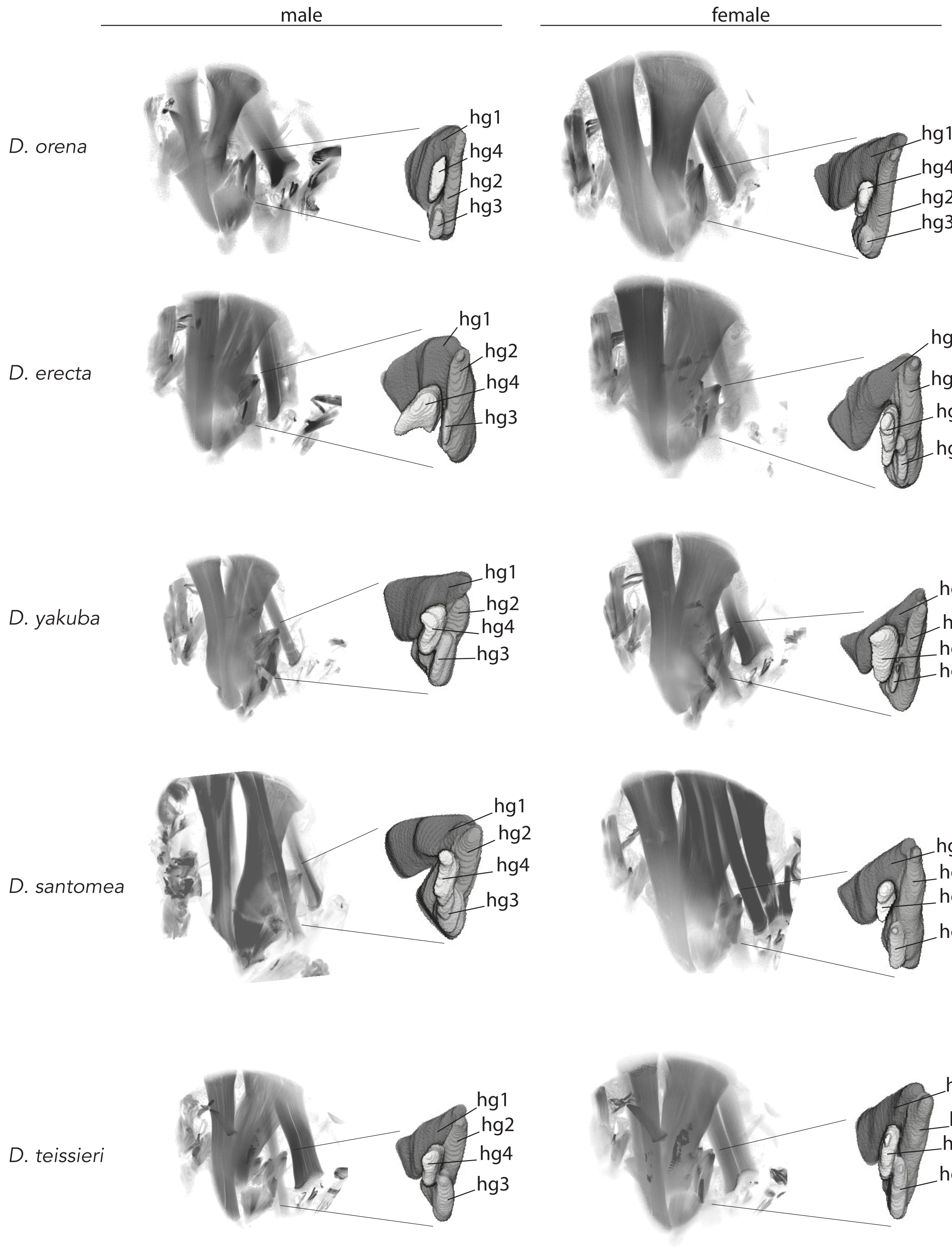
male
female
D. orena
hg1
hg4
hg2
hg3
hg1
hg4
hg2
hg3
D. erecta
hg1
hg2
hg4
hg3
hg1
hg2
hg4
hg3
D. yakuba
hg1
hg2
hg4
hg3
hg1
hg2
hg4
hg3
D. santomea
hg1
hg2
hg4
hg3
hg1
hg2
hg4
hg3
D. teissieri
hg1
hg2
hg4
hg3
hg1
hg2
hg4
hg3

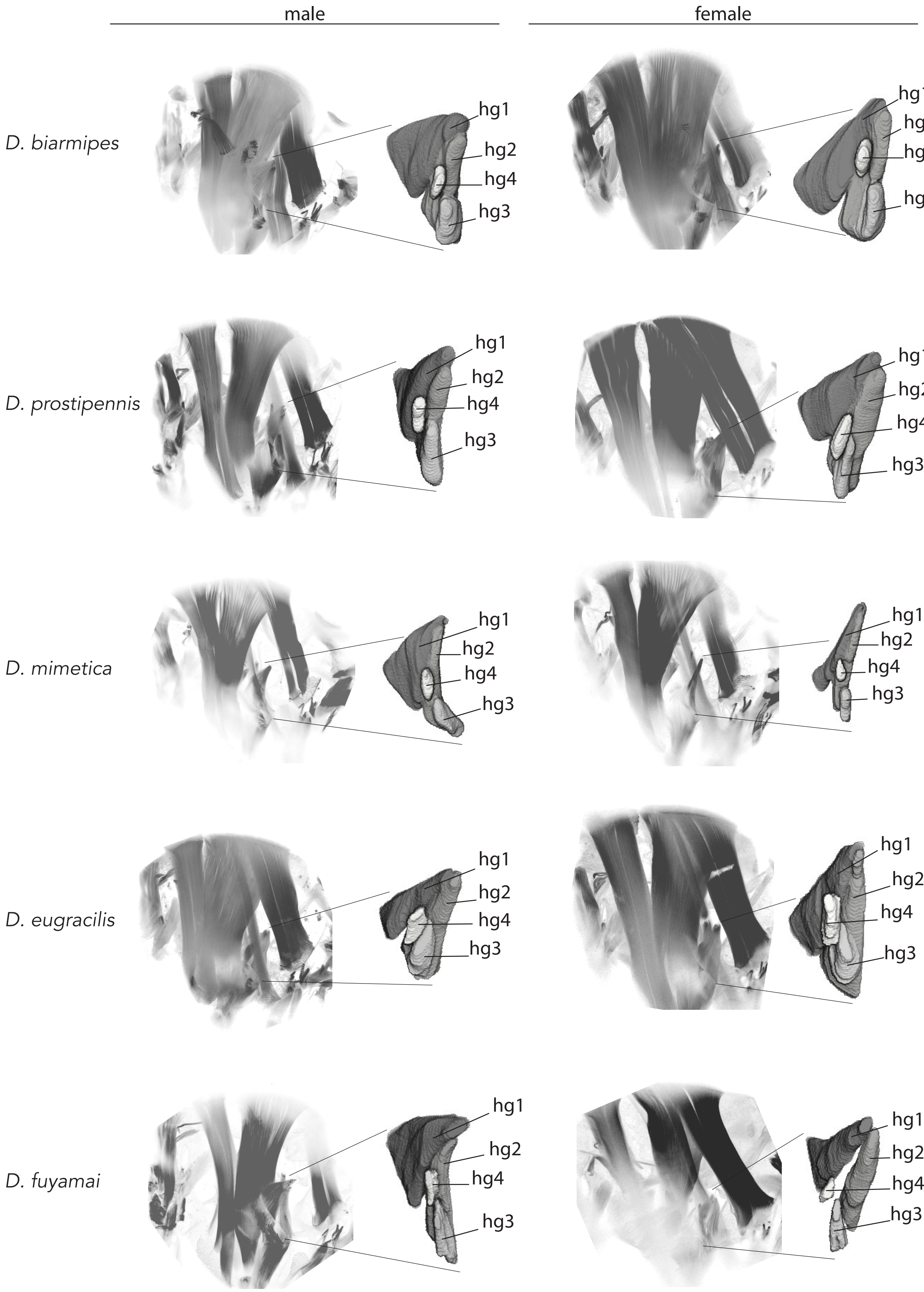
male
female
D. biarmipes
hg1
hg2
hg4
hg3
hg1
hg2
hg4
hg3
D. prostipennis
hg1
hg2
hg4
hg3
hg1
hg2
hg4
hg3
D. mimetica
hg1
hg2
hg4
hg3
hg1
hg2
hg4
hg3
D. eugracilis
hg1
hg2
hg4
hg3
hg1
hg2
hg4
hg3
D. fuyamai
hg1
hg2
hg4
hg3
hg1
hg2
hg4
hg3

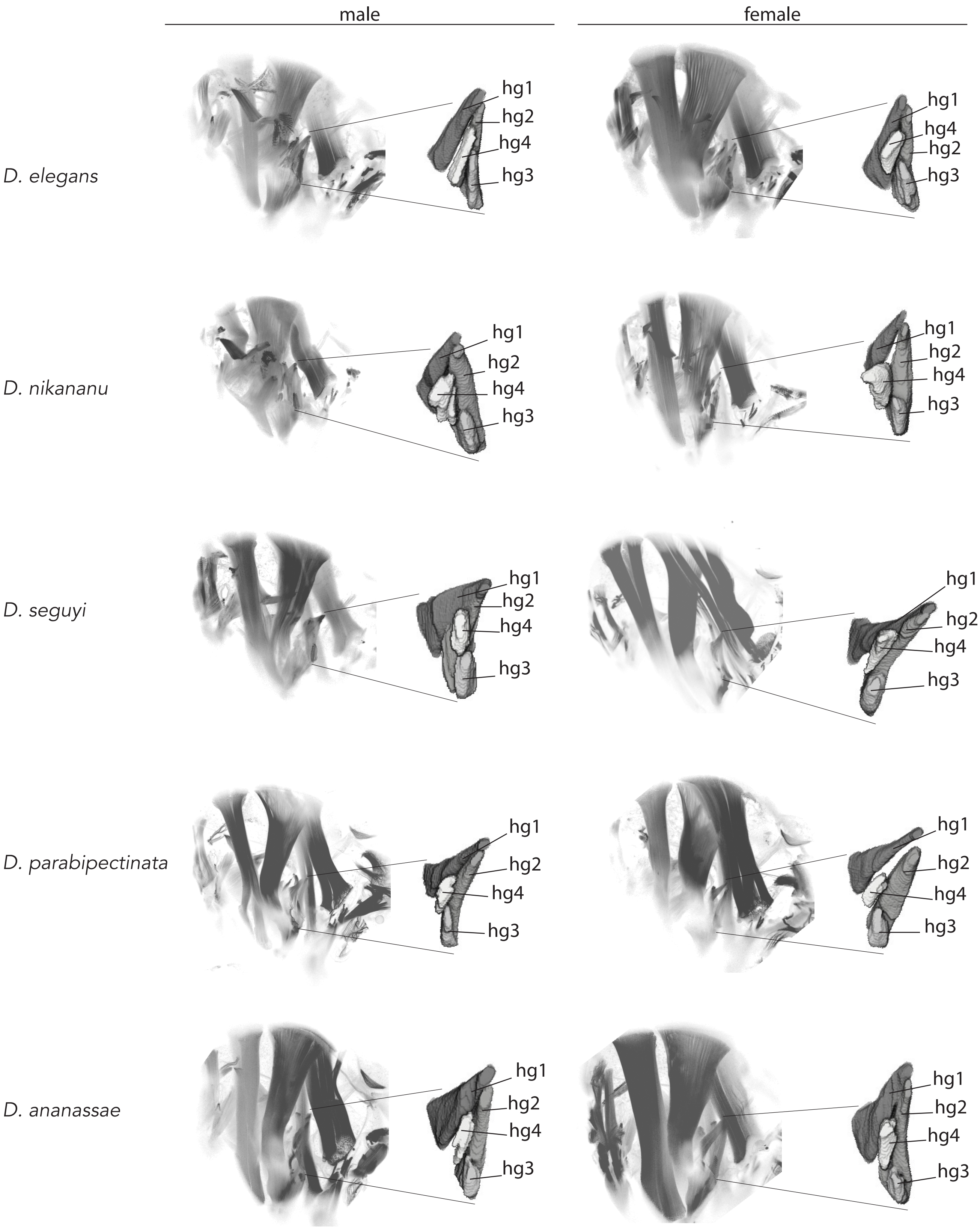
male
female
D. elegans
hg1
hg2
hg4
hg3
hg1
hg4
hg2
hg3
D. nikananu
hg1
hg2
hg4
hg3
hg1
hg2
hg4
hg3
D. seguyi
hg1
hg2
hg4
hg3
hg1
hg2
hg4
hg3
D. parabipectinata
hg1
hg2
hg4
hg3
hg1
hg2
hg4
hg3
D. ananassae
hg1
hg2
hg4
hg3
hg1
hg2
hg4
hg3

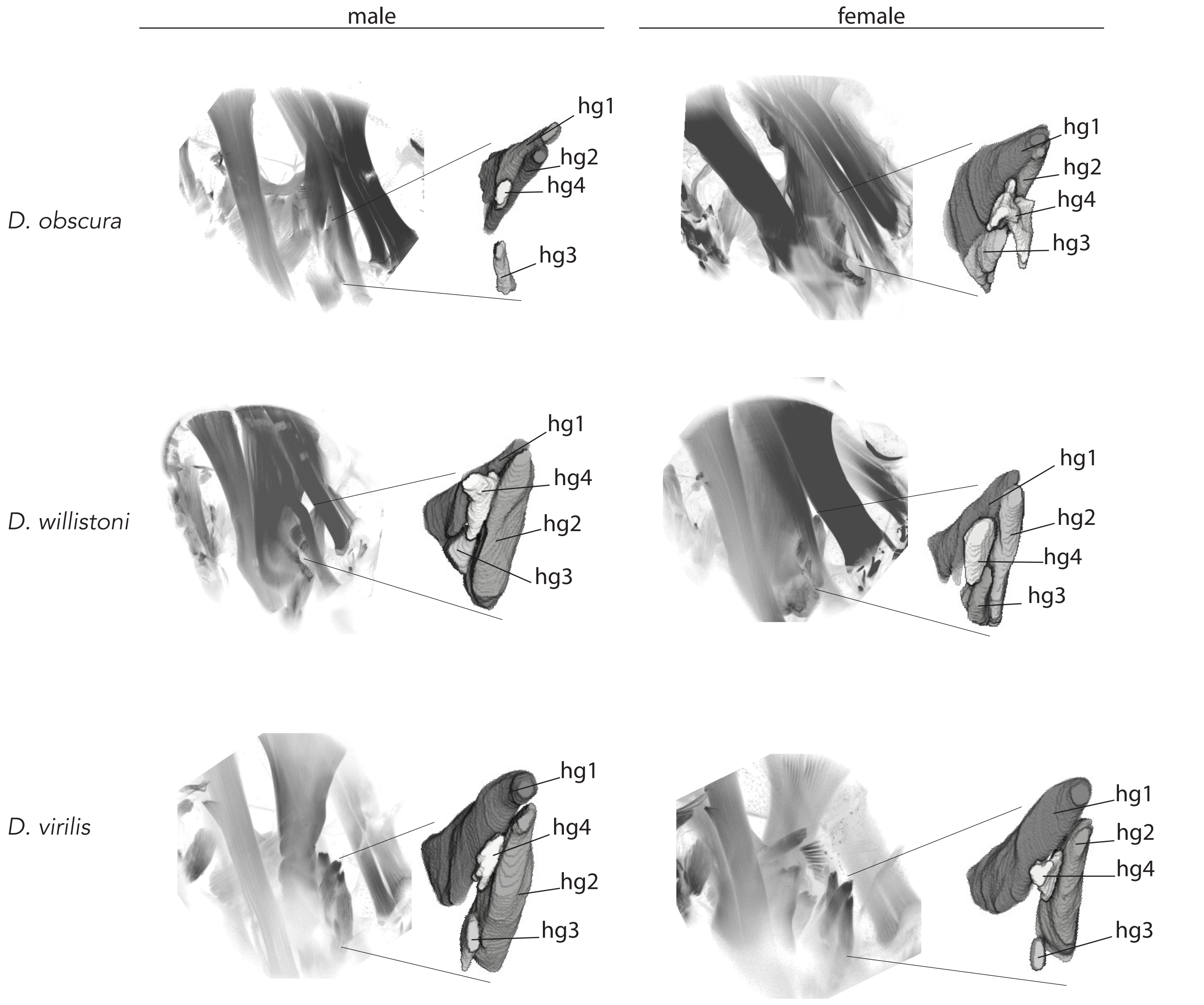

Supplemental Figure 4. Reconstructions of the hg1–4 muscles from males and females of the species used in this study. The relative position and shape of the muscles are generally conserved in males and females across all species. In all images, the anterior side of the thorax is to the left and the dorsal side of the thorax is to the top. Maximum projection image of the confocal slices that include the hg muscles is shown to the left of each panel. Reconstructions on the right were generated in Amira.

Node	Hg1		Hg2			Hg3		
	Prob (mono)	Prob (di)	Prob (mono)	Prob (male enlarged)	Prob (female enlarged)	Prob(mono)	Prob (male enlarged)	Prob (female enlarged)
1	0.702	0.298	0.966	0.017	0.017	0.956	0.022	0.022
2	0.719	0.281	0.978	0.011	0.012	0.970	0.015	0.015
3	0.785	0.215	0.990	0.004	0.006	0.989	0.005	0.005
4	0.843	0.157	0.991	0.002	0.007	0.998	0.001	0.001
5	0.811	0.189	0.999	0.000	0.001	0.999	0.001	0.000
6	0.563	0.437	0.999	0.000	0.000	0.992	0.007	0.001
7	0.161	0.839	0.979	0.017	0.004	0.999	0.001	0.000
8	0.018	0.982	1.000	0.000	0.000	1.000	0.000	0.000
9	0.013	0.987	1.000	0.000	0.000	1.000	0.000	0.000
10	0.086	0.914	1.000	0.000	0.000	0.980	0.000	0.019
11	0.001	0.999	1.000	0.000	0.000	1.000	0.000	0.000
12	0.000	1.000	1.000	0.000	0.000	0.990	0.010	0.000
13	0.141	0.859	0.897	0.085	0.018	0.998	0.002	0.000
14	0.081	0.919	0.435	0.473	0.092	0.993	0.006	0.001
15	0.029	0.971	0.169	0.516	0.315	0.885	0.109	0.006
16	0.579	0.421	1.000	0.000	0.000	0.960	0.038	0.003
17	0.990	0.010	1.000	0.000	0.000	1.000	0.000	0.000
18	0.973	0.027	0.871	0.006	0.124	0.999	0.000	0.000

Supplemental Table 1. Results from the ancestral state reconstruction for hg1–hg3 with the probability of each node being monomorphic or dimorphic. Results for hg4 are not shown, as no dimorphisms were present in the dataset (all nodes are inferred to be monomorphic).

	ADH	amyrel	Ddc	Gphd	Sod	Xdh
Chymomyza amoena	AF059887	AY736544	AY124490	L36961	X61687	AF093217
Chymomyza procnemis	AB026521	-	-	L41252	AF021821	-
Drosophila ananassae	XM001961486	AF024691	-	XM001962674	-	-
Drosophila biarmipes	DQ363229	AF462597	DQ363223	AY098467	-	DQ363235
Drosophila elegans	DQ363230	AF136930	DQ363224	AB032146	-	DQ363236
Drosophila erecta	XM001968861	AF039562	-	XM001968825	AF127156	-
Drosophila eugracilis	AY279326	AF250055	DQ363225	AY098472	-	AY279341
Drosophila ficusphila	DQ363232	AF462600	DQ363226	AB032149	-	DQ363237
Drosophila flavohirta	-	AY733051	-	-	-	-
Drosophila fuyamai	-	-	-	AY098471	-	-
Drosophila gunungcola	AM181670	-	-	AB032145	-	-
Drosophila levii	-	AF491635	-	-	-	-
Drosophila lucipennis	AF459751	AF251138	-	AY098470	-	-
Drosophila lutescens	AY279327	AF491637	-	-	-	AY279343
Drosophila mauritiana	X63953	U96157	-	-	AF127158	-
Drosophila melanogaster	X78384	NM057914	X04661	J04567	X17332	Y00308
Drosophila mimetica	AY279328	AY733058	-	AY098465	-	AY279342
Drosophila nikananu	AF459767	AF251136	-	-	-	-
Drosophila obscura	U90955	AF306713	-	U47881	U47892	-
Drosophila orena	Z00032	U96158	-	DQ167752	AF127155	AY279340
Drosophila parabipectinata	AB194414	AY736489	-	AJ844754	AJ844865	-
Drosophila paralutea	AF459747	-	-	-	-	-
Drosophila prostipennis	AF459748	-	-	AB027282	-	-
Drosophila pseudotakahashii	-	AY736499	-	-	-	-
Drosophila santomea	AY804554	AY736503	-	-	AY804512	AY804550
Drosophila sechellia	XM002035803	AF039558	-	-	AF127157	-
Drosophila seguyi	AF459765	-	-	-	-	-

Drosophila simulans	XM002079432	U96160	AY197770	L41647	X15685	AY279336
Drosophila subelegans	-	AY736507	-	-	-	-
Drosophila takahashii	AF459749	U96161	-	-	-	-
Drosophila teissieri	X54118	AF039557	-	U47809	AF127160	AY279337
Drosophila virilis	XM002057547	XM002048980	AF293749	XM002051332	X13831	AF093215
Drosophila willistoni	XM002066420	AF039560	AF293750	XM002065330	L13281	AF093206
Drosophila yakuba	XM002089270	AF039561	-	XM002089126	AF127159	AY279338
Leucophenga orientalis	AB033638	-	-	-	-	-

Supplemental Table 2. GenBank accession numbers of species used in the phylogenetic analysis. All data was used for the Maximum Likelihood estimation of the phyogeny, and the bolded individuals were the only ones retained on the phylogeny for ancestral state reconstruction.
